# Supplementary material for: Pathophysiological Significance of Neutrophilic Transfer RNA-Derived Small RNAs in Asymptomatic Moyamoya Disease
Source: Cells. 2021 May 1;10(5):1086. doi: 10.3390/cells10051086 (PMC8147334; doi:10.3390/cells10051086)

2D Structure

4043 4071

8mer-1a

```
5'-ttGCATAC-AAGATTTATTGCAATGTGGGa-3' utr
    |  ||  |  ||     :||    :|  |||||||
3'-ucCUUAGGAUUGGCGAU-CUGGUACACCCu-5' tRF
```

16 15 14 13 — 3' Pairing

7 6 5 4 3 2 — Seed

Local AU

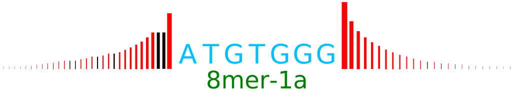

Position

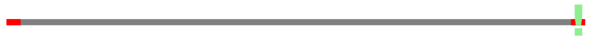

Supplement: Supplementary file 1 [file cells-10-01086-s001.zip › cells-1149161 3rd supplementary materials/Data Supplements/Data Supplement V.zip!/Data supplement 6-mRNAs targets of tsRNAs/tsRNA-Glu-TTC-010/Diagrams/tRF-Glu-TTC-010_vs_ENST00000264414(CUL3).pdf]
